# DNA damage response and preleukemic fusion genes induced by ionizing radiation in umbilical cord blood hematopoietic stem cells

**DOI:** 10.1038/s41598-020-70657-z

**Published:** 2020-08-24

**Authors:** Pavol Kosik, Matus Durdik, Lukas Jakl, Milan Skorvaga, Eva Markova, Gabriela Vesela, Lenka Vokalova, Lucia Kolariková, Eva Horvathova, Katarina Kozics, Igor Belyaev

**Affiliations:** 1 Deparment of Radiobiology, Biomedical Research Center, Cancer Research Institute, Slovak Academy of Sciences, Dubravska cesta 9, 845 05 Bratislava, Slovakia; 2 Deparment of Genetics, Biomedical Research Center, Cancer Research Institute, Slovak Academy of Sciences, Bratislava, Slovak Republic

**Keywords:** Haematological cancer, Paediatric cancer, Haematopoietic stem cells, Apoptosis, DNA damage and repair

## Abstract

There is clear evidence that ionizing radiation (IR) causes leukemia. For many types of leukemia, the preleukemic fusion genes (PFG), as consequences of DNA damage and chromosomal translocations, occur in hematopoietic stem and progenitor cells (HSPC) in utero and could be detected in umbilical cord blood (UCB) of newborns. However, relatively limited information is available about radiation-induced apoptosis, DNA damage and PFG formation in human HSPC. In this study we revealed that CD34+ HSPC compared to lymphocytes: (i) are extremely radio-resistant showing delayed time kinetics of apoptosis, (ii) accumulate lower level of endogenous DNA damage/early apoptotic γH2AX pan-stained cells, (iii) have higher level of radiation-induced 53BP1 and γH2AX/53BP1 co-localized DNA double stranded breaks, and (iv) after low dose of IR may form very low level of BCR-ABL PFG. Within CD34+ HSPC we identified CD34+CD38+ progenitor cells as a highly apoptosis-resistant population, while CD34+CD38− hematopoietic stem/multipotent progenitor cells (HSC/MPP) as a population very sensitive to radiation-induced apoptosis. Our study provides critical insights into how human HSPC respond to IR in the context of DNA damage, apoptosis and PFG.

## Introduction

Over the last few decades, the knowledge of radiation-induced carcinogenesis has significantly progressed. Leukemia, that is: (i) characterized by abnormal proliferation of hematopoietic stem and progenitor cells (HSPC) and (ii) the most common cancer in children^1^, was one of the first cancers connected to ionizing radiation (IR)^2^. Much information has come from epidemiological studies of atomic bomb survivors, medically, occupationally and environmentally exposed persons^3–5^.

One of the most clinically important factors for arising and treatment of leukemia is the presence of preleukemic fusion genes (PFG). The most frequent PFG are MLL-AF4, BCR-ABL, TEL-AML1 and AML1-ETO^6^. While few studies have analyzed induction of PFG by radiation-induced DNA damage in different cell lines and after high doses of ionizing radiation^7–9^, the ability of IR to induce PFG in HSPC has not yet been tested. Of note, our screening of PFG from umbilical cord blood (UCB) samples of healthy donors^10,11^, but also other scientific groups^12–14^, have shown high incidence of PFG, ranging from 1 to 5%. This value significantly exceeded the incidence of PFG+ leukemia and suggested that only those PFG, which arise in specific HSPC populations, may result in overt leukemia after accumulation of further mutations^10^. Till now, only few studies have comparatively analyzed apoptosis and DNA damage in HSPC and lymphocytes^15–19^ and even less compared apoptotic and DNA damage response in different populations of HSPC^15^.

In this study we examined DNA damage and apoptosis in UCB lymphocytes and different subsets of CD34+ HSPC in response to IR. Concurrently, we analyzed the effect of IR on the most frequent PFG in acute lymphoblastic (ALL) and myeloid (AML) leukemia, specifically TEL-AML1, MLL-AF4, BCR-ABL, and AML1-ETO. Here, we show in line with our previously published data^18^ higher apoptosis-resistance and late onset of apoptosis in HSPC. We identified CD34+CD38+ progenitors as an extremely resistant cell population and, in contrary, CD34+CD38− HSC/MPP as a population very sensitive to endogenous and radiation-induced apoptosis. Analysis of γH2AX fluorescence/early apoptotic pan-stained cells confirmed data from apoptosis also showing lower level of endogenous DNA damage in HSC/MPP (CD34+CD38−) and progenitors (CD34+CD38+) compared to lymphocytes. We also detected higher level of radiation-induced 53BP1 and 53BP1/γH2AX DNA repair foci in CD34+ HSPC compared to lymphocytes. We revealed induction of BCR-ABL PFG after low doses of IR, while high doses were not effective most probably due to apoptotic elimination of damaged PFG+ cells.

## Results

### Apoptosis

#### CD34+ HSPC vs. CD34− lymphocytes

**Figure 1:**
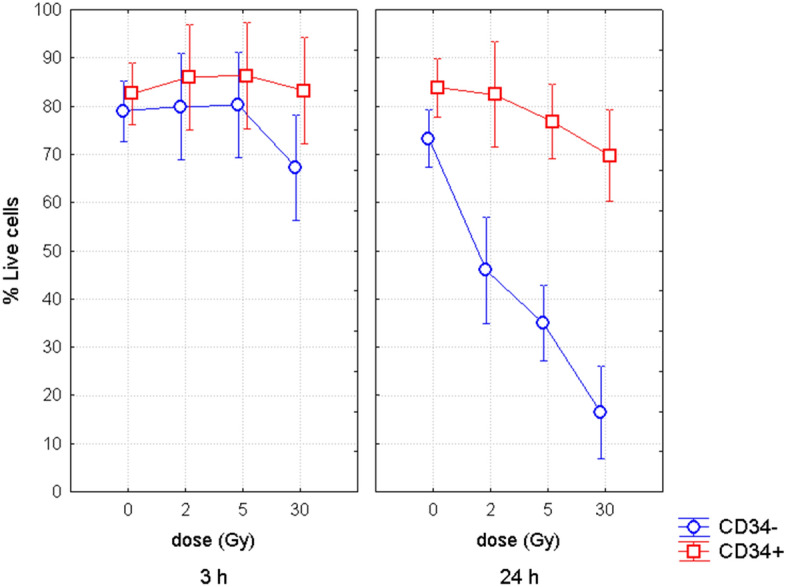
Apoptosis in CD34− lymphocytes and CD34+ HSPC. Figure shows percentage of live cells in CD34-lymphocytes and CD34+ HSPC at different time points post-irradiation with the dose of 0, 200, 500, and 3,000 cGy. Mean value from at least 3 independent experiments and 95% confidence interval is shown in each data point.

Standard markers for early and late cellular apoptosis, Annexin-V and 7AAD, respectively, were used to analyze apoptosis in UCB CD34− lymphocytes and CD34+ HSPC at 3 h and 24 h post-irradiation (Fig. 1). Applying multifactorial ANOVA to all data obtained in both populations, we found that apoptosis in UCB cells depended on dose and time after irradiation (p < 0.000001 and < 0.000001, respectively). In addition, multifactorial ANOVA revealed statistically significant effect of membrane marker (p < 0.000001) indicating different apoptotic response of HSPC and lymphocytes.

Next we analyzed data in separate cell populations, CD34+ and CD34−, by applying univariate ANOVA. We found significant decrease in percentage of live CD34− lymphocytes from 3 to 24 h post-irradiation (p < 0.000001), while no statistically-significant decrease was revealed for CD34+ HSPC (p = 0.09) suggesting lower radio-sensitivity of these cells. Dose-dependent apoptosis was detected 3 h after irradiation in neither CD34− nor CD34+ cells (p = 0.1 and 0.9, correspondently). However, 24 h after irradiation, we detected a clear dose-dependent decrease of live CD34− lymphocytes (p = 0.000001), while, again, no dose-dependence was seen in CD34+ HSPC (p = 0.17). Significant decrease of live CD34− cells was observed 24 h after irradiation with 2 Gy, 5 Gy, 30 Gy (Fischer LSD, p = 0.00005, < 0.000001, < 0.000001, respectively), although only the dose of 30 Gy induced apoptosis in CD34+ cells (Fischer LSD, p = 0.015). Higher apoptosis was revealed in CD34− cells compared to CD34+ cells 3 h post-irradiation with the dose of 30 Gy (Fischer LSD, p = 0.04). In general, 24 h post-irradiation, apoptosis was clearly higher in CD34− cells compared to CD34+ cells regardless the dose of irradiation (0, 2, 5 and 30 Gy, Fischer LSD, p = 0.016, 0.00002, < 0.000001, < 0.000001, respectively).

Thus, altogether data clearly showed that CD34+ HSPC are extremely resistant to radiation and might have a delayed kinetics of apoptosis compared to CD34− lymphocytes.

#### HSC/MPP vs. progenitors and vs. CD34−

**Figure 2:**
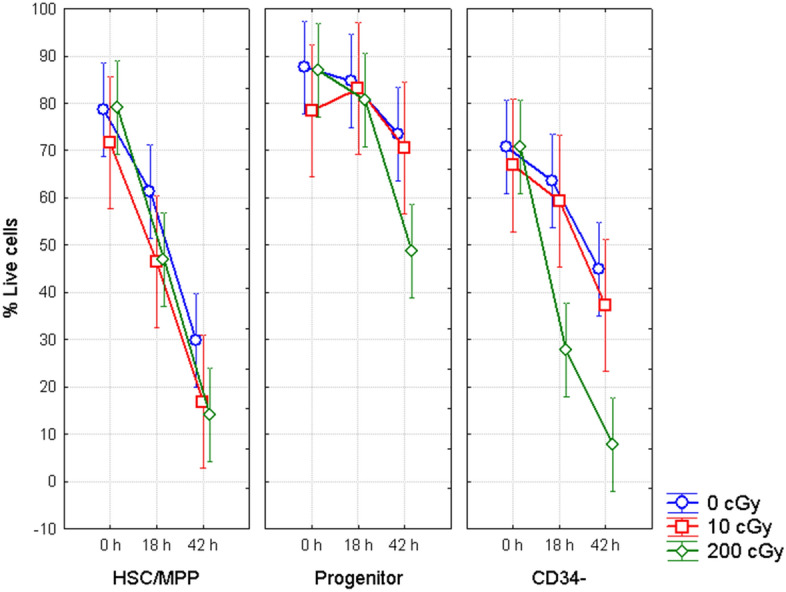
Apoptosis in CD34+CD38+ progenitors and CD34+CD38− HSC/MPP. Figure shows percentage of live cells in CD34− lymphocytes, CD34+CD38+ progenitors and CD34+CD38− HSC/MPP at different time points post-irradiation with doses 0, 10, and 200 cGy. Mean value and 95% confidence interval is shown from 6 samples for 200 cGy, and 3 samples for 10 cGy.

Deeper investigation of the apoptosis time kinetics up to 42 h post-irradiation was carried out in the set of experiments where irradiated cells were further gated into CD34+CD38− HSC/MPP, CD34+CD38+ progenitor cells, and CD34− lymphocytes using appropriate CD markers. In addition to high dose of 2 Gy, in these experiments we irradiated cells with the dose of 10 cGy to assess the inducibility of apoptosis by low doses of IR. Comparison of HSC/MPP with progenitor cells and lymphocytes showed that not whole population of CD34+ HSPC is resistant to radiation-induced apoptosis (Fig. 2, Supplementary Figure 1). Higher sensitivity to radiation was detected in CD34+CD38− HSC/MPP (in average 9.1% of the whole CD34+ HSPC), which contains the most primitive HSC. This higher sensitivity of HSC/MPP to apoptotic process compared to CD34+CD38+ progenitor cells was observed at 18 h after irradiation with doses of 10 cGy and 2 Gy (Fischer LSD, p = 0.0003 and 0.000006, correspondently), and was even more prominent at 42 h (ANOVA, p < 0.000001). HSC/MPP population also showed higher sensitivity to endogenous apoptosis at 18 h and 42 h compared to progenitor cells (Fisher LSD, p = 0.0012 and 0.0000001, respectively). Similarly, HSC/MPPs were more sensitive to endogenous apoptosis as compared with lymphocytes. Indeed, there were significantly lower percentage of HSC/MPP live cells in comparison to lymphocytes 42 h after sham irradiation and irradiation with 10 cGy (Fischer LSD, p = 0.03, 0.04, respectively). The dose of 10 cGy induced apoptosis neither 18 h nor 42 h after irradiation in any of the cell populations studied. Thus, similar to sham-irradiated cells, apoptosis was predominantly endogenous in the samples irradiated with 10 cGy. On the other hand, HSC/MPP were more resistant to the radiation-induced apoptosis compared to lymphocytes 18 h after irradiation with 2 Gy (Fischer LSD, p = 0.008). Since the level of endogenous apoptosis was relatively high in HSC/MPP after 42 h, there was no difference in the radiation-induced apoptosis between HSC/MPP and lymphocytes 42 h after irradiation with 2 Gy.

Next we compared results for CD34− lymphocytes and CD34+ HSPC (Supplementary Figure 2) by combining the data from HSC/MPP and progenitors shown in Fig. 2 with the data presented in Fig. 1. The results of both experimental sets were very consistent showing lower endogenous and radiation-induced apoptosis of CD34+ HSPC compared to CD34− lymphocytes. The same as in the first set of experiments (Fig. 1), lower radiation-induced apoptosis of CD34+ HSPC was revealed after irradiation with the dose of 200 cGy and longer incubation time, 18 h and 42 h (Fischer LSD, p = 0.000001 and 0.000001, respectively). Moreover, lower endogenous apoptosis was also observed in CD34+ HSPC at 0 h, 18 h and 42 h (Fischer LSD, p = 0.008, 0.003, and 0.0002, respectively).

The obtained results revealed that the differences in apoptotic response observed between CD34+ HSPC and CD34− lymphocytes (Fig. 1, Supplementary Figure 2)^18^, are in fact caused by a higher resistance of progenitor cells (Fig. 2), which represent about 90% of CD34+ HSPC.

### Evaluation of DSB

**Figure 3:**
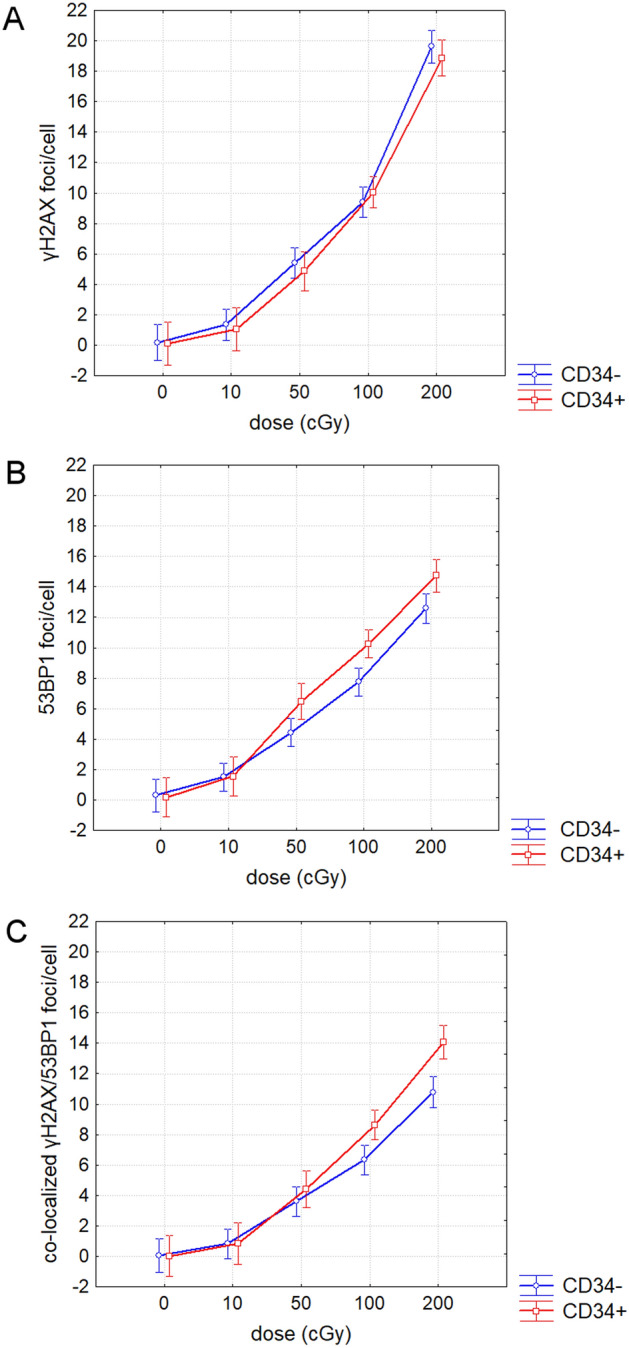
Dose–response for the γH2AX (**A**), 53BP1 (**B**) and their co-localization γH2AX/53BP1 (**C**) in CD34+ and CD34− cells 0.5 h after irradiation with γ-rays. Mean value and 95% confidence interval is shown.

Fresh UCB mononuclear cells (MNC) were immune-magnetically separated into CD34− lymphocytes and CD34+ HSPC, and then irradiated by γ-rays with doses of 0, 10, 50, 100, and 200 cGy. DNA repair foci were analyzed 0.5 h post-irradiation (Fig. 3). We observed a dose-dependent increase of γH2AX and 53BP1 foci and also their co-localization in both CD34− and CD34+ cells (ANOVA, p < 0.0000001 for all endpoints). No difference was revealed in the level of endogenous γH2AX, 53BP1 foci and co-localization between CD34− and CD34+ (Fisher LSD, p = 0.93, 0.88, and 0.97, respectively). However, we found increased level of IR-induced 53BP1 foci and γH2AX/53BP1 co-localization in CD34+ HSPC compared to CD34− lymphocytes (ANOVA p = 0.0002 and 0.0005, respectively), while no increase for IR-induced γH2AX foci (ANOVA, p = 0.59). In line with analysis of variance, Fisher LSD test has shown no increased level of γH2AX foci in CD34+ compared to CD34− cells at any dose of 50, 100, and 200 cGy (p = 0.73, 0.52, 0.36, and 0.35, respectively). The same test revealed higher level of 53BP1 foci at the doses of 50, 100, and 200 cGy (p = 0.008, 0.0003, and 0.004, respectively) and higher γH2AX/53BP1 co-localization at the doses of 100 and 200 cGy (p = 0.00047 and 0.00044, respectively). Because kinetics of H2AX histone phosphorylation and re-localization of 53BP1 to the location of DNA damage might be different^20^, we also analyzed % of γH2AX and 53BP1 foci presented in co-localized foci. The % of co-localized γH2AX and 53BP1 in non-irradiated CD34− cells was comparable to that in CD34+ (Fisher LSD, p = 0.3 and 0.9, respectively) and reached in average 0.31% *vs* 0.23% for γH2AX and 0.16% vs. 0.16% for 53BP1. In irradiated CD34− cells % of co-localized 53BP1 foci at all doses of γ-rays (10, 50, 100, 200 cGy) was comparable to that in CD34+ cells (Fisher LSD, p = 0.66, 0.16, 0.82, and 0.11, respectively), while % of γH2AX co-localized foci slightly increased in CD34+ cells compared to CD34− cells from the dose of 50 cGy (Fisher LSD, p = 0.05, 0.004, 0.004, and 0.011, respectively). Our results show the difference in the signaling pathway of DNA damage response between CD34+ HSPC and CD34− lymphocytes, which is likely caused by different time kinetics of γH2AX and 53BP1 proteins.

### Measurement of γH2AX fluorescence

**Figure 4:**
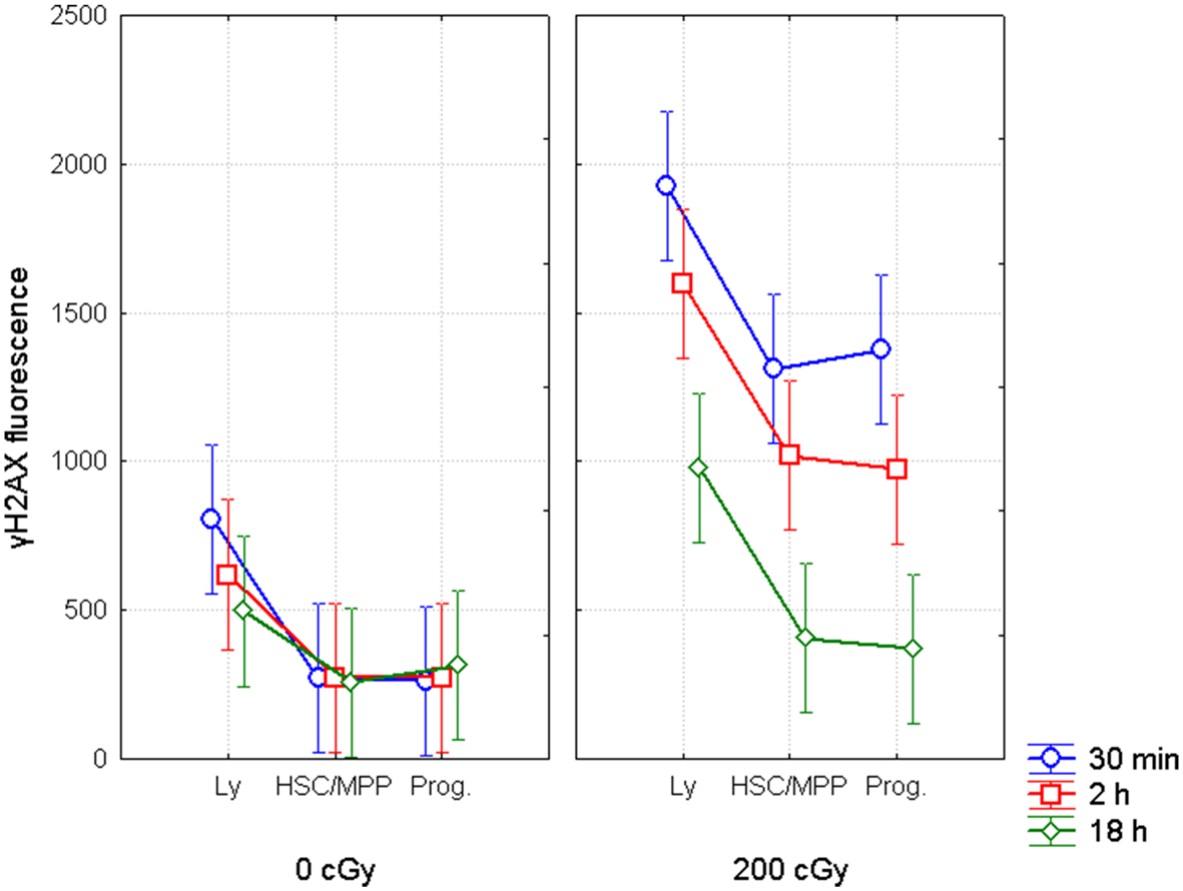
γH2AX fluorescence in different populations of HSPC and in lymphocytes. Total fluorescence of γH2AX in HSC/MPP (CD34+38−), progenitor cells (CD34+38+) and lymphocytes (CD34−) analyzed by flow cytometry at different time points after irradiation with the dose of 0 (left panel) and 200 cGy (right panel). Figure shows the mean values of fluorescence from 3 independent experiments with 95% confidence interval.

Total γH2AX fluorescence as an indicator of the amount of DNA double strand breaks and early apoptotic γH2AX pan-stained cells^21^ was analyzed by flow cytometry in CD34− lymphocytes CD34+CD38− HSC/MPP and CD34+ CD38+ progenitor cells 30 min, 2 h, and 18 h after 200 cGy of γ-rays (Fig. 4).

In line with our previously published data^11,18^, endogenous γH2AX fluorescence at 30 min was lower in CD34+CD38− HSC/MPP and CD34+CD38+ progenitor cells compared with CD34− lymphocytes (Fisher LSD, p = 0.004 and 0.004, respectively). This difference disappeared during further cultivation of cells (Fig. 4). We also did not see any difference in endogenous level of γH2AX fluorescence between CD34+CD38− HSC/MPP, and CD34+CD38+ progenitors at all time points (Fisher LSD, p = 0.96, 0.1, and 0.73).

In all irradiated samples, a time-dependent decrease of γH2AX fluorescence was found in all three cell populations, such as: lymphocytes, HSC/MPP and progenitors (ANOVA, p = 0.04, 0.005, and 0.006, respectively). Analysis of the data also showed that IR exposed CD34− lymphocytes had higher level of γH2AX fluorescence than IR exposed HSC/MPP or progenitors at 30 min, 2 h, and 18 h [Fisher LSD, (30 min) p = 0.001 or 0.003, (2 h) 0.002 or 0.001, (18 h) 0.002 or 0.001, respectively]. No difference was found between HSC and progenitors at all analyzed time points (Fisher LSD p = 0.71, 0.78, and 0.84, respectively).

To conclude, our results suggest lower accumulation of endogenous and IR-induced DNA damage/early apoptosis in CD34+CD38− HSC/MPP and CD34+CD38+ progenitor cells compared to CD34− lymphocytes. Along with our data on evaluation of DSB, which did not confirmed higher level of IR-induced γH2AX foci in CD34− lymphocytes compared to CD34+ HSPC, these results strengthens suggestion, that increased γH2AX fluorescence in IR-exposed lymphocytes was accounted for higher fraction of early apoptotic γH2AX pan-stained cells.

### Comet assay

**Figure 5:**
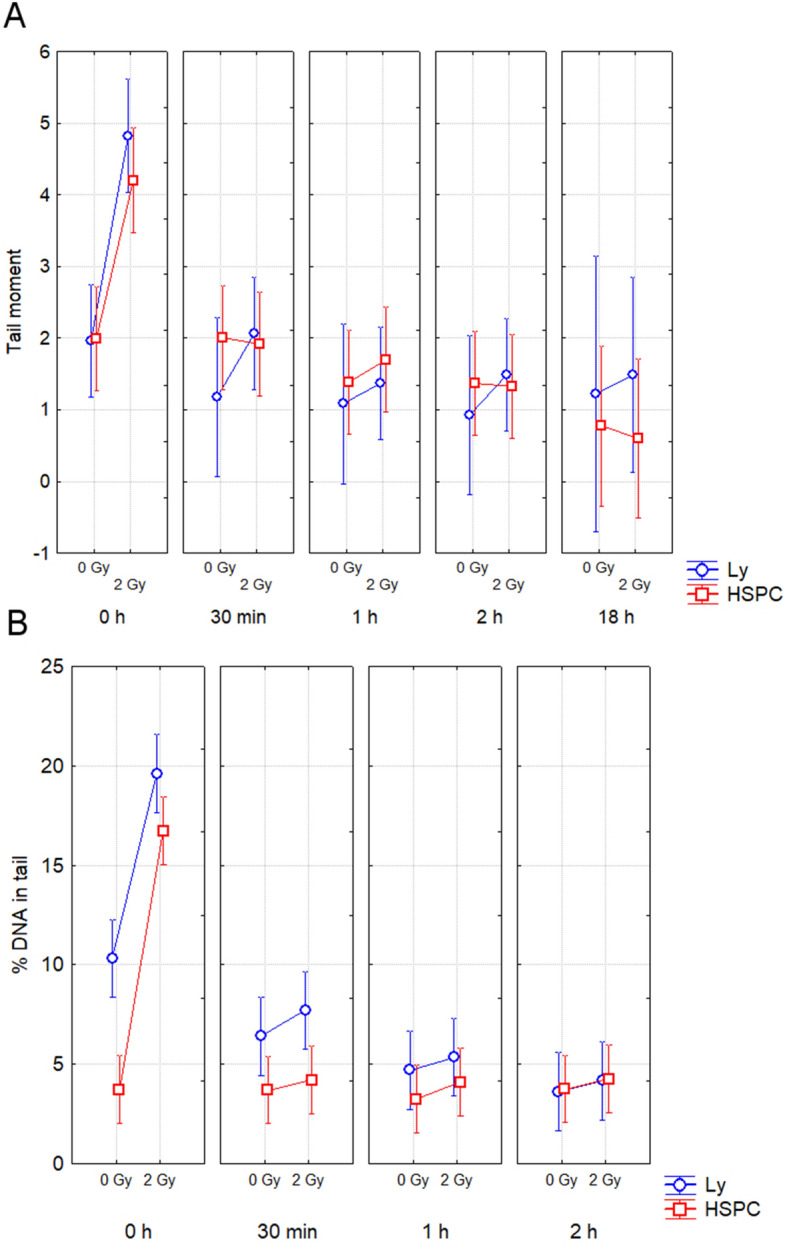
Evaluation of DNA damage by comet assay. DNA damage analyzed at 0 h, 30 min, 1 h and 2 h after irradiation. Mean value and 95% confidence interval is shown for each time points. (**A**) By the neutral comet assay we analyzed samples from 15 probands. (**B**) Of them, 7 probands have been chosen for testing by alkaline comet assay.

Freshly collected and immune-magnetically separated cells were exposed to 2 Gy of γ-rays and analyzed by neutral and alkaline comet assays 0 min, 30 min, 1 h, 2 h, and 18 h after irradiation (Fig. 5A,B). Increased DNA damage was observed immediately (0 h) after irradiation in CD34+ and CD34− cells by both methods (Fisher LSD, neutral comet: p = 0.00005, 0.000002 and alkaline comet: p = 0.000001, 0.00001, respectively). At the later time points (30 min, 1 h, 2 h, 18 h) DNA damage was gradually repaired and finally decreased to endogenous level. Neutral comet assay revealed differences in neither endogenous nor IR-induced DNA damage between CD34+ and CD34− cells. However, using alkaline comet assay we found significantly lower level of endogenous DNA damage in CD34+ HSPC (ANOVA, p = 0.002), which was observed both at 0 h and 30 min (Fisher LSD, p = 0.000008 and 0.04, respectively). These results supported the data from analysis of γH2AX fluorescence and apoptosis (Figs. 2 and 4). Lower endogenous DNA damage in CD34+ cells was not evident 1 h and 2 h post-irradiation (Fisher LSD, p = 0.46 and 0.91, respectively). Taking into account endogenous DNA damage evaluated by alkaline comet assay, no difference in response to radiation was found between cell types (ANOVA, p = 0.43).

Finally, the results show the same radiation-induced DNA damage response in CD34+ HSPC and CD34− lymphocytes as analyzed by neutral comet assay. However, lower level of endogenous DNA damage was detected in CD34+ HSPC by alkaline comet assay.

### PFG induction

**Figure 6:**
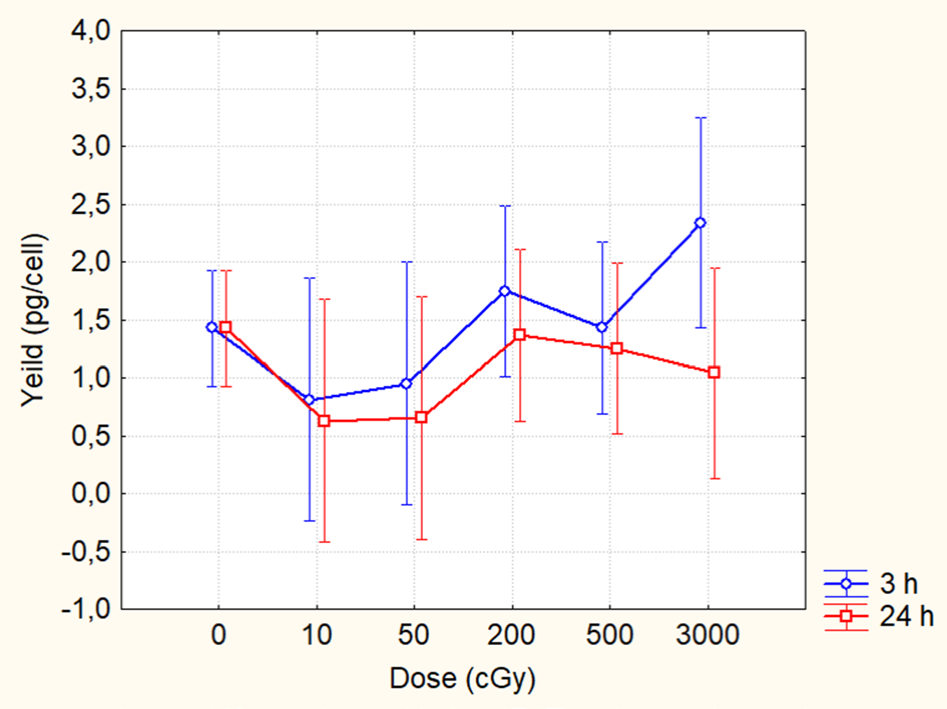
RNA yield from irradiated and non-irradiated UCB samples. RNA yield measured by the Nanodrop 1,000 spectrophotometer at 3 h and 24 h after exposure of samples to 0, 10, 50, 200, 500, and 3,000 cGy of γ-rays. Mean value and 95% confidence interval is shown from 3 samples for 0, 10 and 50 cGy, from 6 samples for 200 and 500 cGy, and from 4 samples for 3,000 cGy at 3 and 24 h.

Using RT-qPCR, we analyzed five groups of UCB samples for induction of BCR-ABL, TEL-AML1, MLL-AF4, and AML1-ETO PFG. Individual groups were formed due to insufficient amount of cryopreserved cells for analysis of all four chosen PFG in a single experiment. As it is shown in Table 1, some groups were tested only for one translocation, others for 2 or 3 translocations. Depending on the group, the cells were exposed to 10, 50, 200, 500, 1,000, or 3,000 cGy of γ-rays. RNA was isolated at 3 h and 24 h post-irradiation. The yield of RNA *per* cell varied between 0.17 and 5.34 pg (Fig. 6). We did not see any significant effect of dose and time on RNA yield *per* cell (ANOVA, p = 0.11, 0.23, respectively). While the RNA yield *per* cell slightly decreased with the time post-irradiation, no statistically significant correlation between total transcription and apoptosis was found (R = 0.24, p = 0.06, Spearman). Analysis by RT-qPCR revealed 32 positive samples out of 180 (17.7%). Of them 18 samples were positive for BCR-ABL, 2 for TEL-AML1 and 12 for MLL-AF4 PFG. We did not observe any positive signal for AML1-ETO fusion gene. By sequencing analysis we excluded 7 false positive samples (5 BCR-ABL and 2 MLL-AF4) obtained by RT-qPCR (Table 1). Finally we got 13/30 positive samples for BCR-ABL (43%), 2/60 for TEL-AML1 (3.3%), 10/60 for MLL-AF4 (16%) and 0/30 for AML1-ETO (0%).

**Table 1:** PFG induction by γ-rays.

No. of probands	Time-dose	TEL-AML1 positive	MLL-AF4 positive	BCR-ABL positive	AML1-ETO positive
Group 1 3	3 h-0 Gy	0	1	2	NA
	3 h-0.1 Gy	0	1	3	NA
	3 h-0.5 Gy	0	0	0	NA
	24 h-0.1 Gy	0	0	2	NA
	24 h-0.5 Gy	0	2	1	NA
Control		0	1	2	NA
Irradiated (*3/24 h*), all		(0/0), 0	(1/2), 3	(3/3), 6	NA
Group 2 6	3 h-0 Gy	0	2	NA	NA
	3 h-2 Gy	0	1	NA	NA
	3 h-5 Gy	0	**1/0**	NA	NA
	24 h-2 Gy	0	**1/0**	NA	NA
	24 h-5 Gy	0	0	NA	NA
Control		0	2	NA	NA
Irradiated (*3/24 h*), all		(0/0), 0	(1/0), 1	NA	NA
Group 3 3	3 h-0 Gy	NA	NA	**3/2**	0
	3 h-2 Gy	NA	NA	0	0
	3 h-5 Gy	NA	NA	**2/1**	0
	24 h-2 Gy	NA	NA	**2/0**	0
	24 h-5 Gy	NA	NA	**3/2**	0
Control		NA	NA	2	0
Irradiated (*3/24 h*), all		NA	NA	(1/2), 3	(0/0), 0
Group 4 1	3 h-0 Gy	0	0	NA	0
	3 h-10 Gy	0	0	NA	0
	24 h-10 Gy	0	0	NA	0
Control		0	0	NA	0
Irradiated (*3/24 h*), all		(0/0), 0	(0/0), 0	NA	(0/0), 0
Group 5 4	3 h-0 Gy	0	1	NA	0
	3 h-30 Gy	1	2	NA	0
	24 h-30 Gy	1	0	NA	0
Control		0	1	NA	0
Irradiated (*3/24 h*), all		(1/1), 2	(2/0), 2	NA	(0/0), 0
Control in total		0	4	4	0
Irradiated in total (*3/24 h*), all		(1/1), 2	(4/2), 6	(4/5), 9	(0/0), 0

The table shows individual experiments, which are grouped by the number of enrolled donor’s samples (3, 6, 3, 1, 4). Cells were irradiated by γ-rays (10, 50, 200, 500, 1,000, 3,000 cGy) and screened for TEL-AML1, MLL-AF4, BCR-ABL, AML1-ETO PFG by RT-qPCR 3 and/or 24 h post-irradiation. The positively tested samples were validated by sequencing. NA—not analyzed; X/Y—before sequencing/after sequencing (in bold); (X/Y)—positivity at 3/24 h (in italics).

While some trend of higher incidence of PFG in irradiated samples was observed taking all 5 groups together (Table 1), neither dose nor time after irradiation was a significant factor for PFG induction in any of 5 analyzed groups or in all groups together (ANOVA, p > 0.05). The positivity of BCR-ABL in IR-induced samples was 2.25-fold higher than in sham-irradiated controls (9 vs. 4), while no dose–response was found. Positivity for MLL-AF4 increased slightly from 4 in control to 6 in irradiated samples, while no dose–response was observed, as well. Positivity of TEL-AML1 increased from 0 in non-irradiated controls to 2 in samples irradiated at 30 Gy. As far as low and high doses may induce different response, we also analyzed the low doses (≤ 50 cGy) and the high doses (≥ 200 cGy) separately. The amount of BCR-ABL positive samples increased from 2 in controls to 6 in samples irradiated with low doses (ANOVA, p = 0.021). The other PFGs showed statistically significant induction for neither low nor high doses. We also did not see any difference in the number of PFG copies between positive control and positive irradiated samples, independently on the dose and the type of PFG (T-test, p > 0.05). In conclusion, while the obtained results did not show any dose-dependent induction of PFG, they, however, suggested that BCR and ABL genes are the most suitable partners to form PFG in response to IR, specifically at the low doses as 10 and 50 cGy.

## Discussion

In this study we analyzed apoptosis, DNA damage and PFG induction in UCB CD34+ HSPC and lymphocytes after exposure to ionizing radiation.

Our results showed delayed apoptosis of HSPC, mainly caused by the higher resistance of CD34+CD38+ progenitor cells (about 90% of CD34+ HSPC) to endogenous and radiation induced apoptosis. These results are partially consistent with our previously published studies^11,18^, but the present study goes into deeper details by analyzing progenitor cells and HSC/MPP separately. In addition, we studied apoptosis at low doses of 10 and 50 cGy, and also after prolonged incubation time till 42 h.

The study by Milyavsky et al. was the first one focused on the process of apoptosis in different subsets of CD34+ HSPC^15^. In this study IR-irradiated HSC (Lin-CD34+CD38−CD45RA-CD90+) and MPP (Lin-CD34+CD38−CD45RA-CD90−) populations showed significantly higher proportion of apoptotic cells than irradiated progenitor cells (Lin-CD34+CD38+). These results were consistent with our results, showing high sensitivity to apoptosis in the subset of CD34+CD38− HSC/MPP. Moreover, for the first time, our study compared apoptosis of HSC/MPP and progenitor cells with differentiated lymphocytes. Interestingly, while HSC/MPP cells were much more sensitive to endogenous apoptosis than lymphocytes, they did not reveal higher radio-sensitivity. Such resistance of progenitor cells to radiation-induced and endogenous apoptosis could be due to higher expression of anti-apoptotic proteins^22–25^. Even though abovementioned gene expression studies were performed only on the CD34+ HSPC, the results could also be valid for progenitor cells, since they account for majority (about 90%) of CD34+ population. Besides the study by Milyavsky and our study, Kraft et al. also analyzed apoptosis, however, they compared cell cycle-activated peripheral blood (PB) HSPC and lymphocytes^16^. Their results did not show any difference between HSPC and lymphocytes. This eventual inconsistency could be caused by different condition of cell cultivation (i.e.: (i) growth factor stimulated vs. non- stimulated cells; (ii) HSPC and lymphocytes were cultivated together *vs* separately).

Only few reports provided experimental results dealing with DNA damage response by measuring DNA repair foci in HSPC or their populations. Our data correlate with the results of some of these studies. Vandevoorde et al. quantified co-localizing γH2AX/53BP1 foci in UCB CD34+ HSPC and the majority fraction of human lymphocytes, T-lymphocytes^17^. Similarly to our study, they observed higher level of co-localized γH2AX/53BP1 foci in HSPC compared to lymphocytes. Kraft et al.^16^ observed an increased level of 53BP1 foci in CD34+ enriched cells and no difference in the level of radiation-induced γH2AX foci between cell cycle activated peripheral blood (PB) HSPC and PB lymphocytes. Vasilyev et al., who irradiated CD133+ UCB cells, which contain majority of HSPC, observed a higher level of 53BP1 foci in CD133+ cells, while also as in our study, no difference in the level of γH2AX foci^19^. Milyavsky et al. did a more detailed research by analyzing DNA damage response in sorted and sub-sequentially irradiated populations of CD34+ HSPC marked as HSC (Lin-CD34+CD38−CD45RA-CD90+), multipotent progenitors (MPP) (Lin-CD34+CD38−CD45RA-CD90−), and progenitor cells (Lin-CD34+CD38+)^15^. This group observed a higher level of radiation-induced γH2AX foci in a combined HSC/MPP population compared to progenitors, suggesting delayed DNA repair process in more primitive cell population. There is also one publication that did not support the aforementioned data of Milyavsky et al. but partially support our data on difference between CD34+ HSPC and CD34− lymphocytes^26^. Significantly, this study has shown a nearly identical decline of γH2AX-foci levels in CD34+CD38− , CD34+CD38+ and CD34− cells after exposure to irradiation.

From all aforementioned papers, comet assay was used only in the study by Milyavsky et al.^15^. These authors measured DNA damage by neutral comet assay after dose of 15 Gy. Significantly delayed DNA repair was seen in combined HSC/MPP than in the progenitor population. That result did not fit our result from neutral comet assay, although we used therapeutic dose of 2 Gy. Besides that, HSPC and lymphocytes were not compared by Milyavsky et al.^15^.

Similar to current study, Kraft et al. analyzed γH2AX fluorescence by flow cytometry, too. Contrary to our results obtained by the same method, they found neither lower level of endogenous nor IR-induced DNA damage in cell-cycle activated HSPC as compared to lymphocytes. We suppose that lower accumulation of endogenous damage in HSPC compared to lymphocytes in our experiment could be accounted for the lower level of ROS as was observed in CD34+ HSPC^27–30^. On the other hand, we could not exclude the possibility that lower endogenous and IR-induced γH2AX fluorescence in CD34+ HSPC was caused by higher percentage of early apoptotic γH2AX pan-stained lymphocytes^21,31^.

In the next part of this study we analyzed induction of PFG in mononuclear UCB cells. Although few groups showed induction of PFG after high doses of ionizing radiation (100 Gy) in different cell lines^7–9^, PFG induction in UCB cells after low and therapeutic doses was not investigated. Quina et al. reported that PML-RARα was not induced by 10 Gy of γ-rays in lymphoid IM9 cell line^9^. As opposed to PML-RARα, BCR-ABL was detected with higher frequency in RNA extracted from HL60 cells 48 h post-irradiation with 100 Gy of X-rays^7,8^. *AML1-ETO* induction by γ-rays was observed with higher frequencies in KG1 (25 × 10^–7^) than in HL60 (5 × 10^–7^) cancer cell line^7^. While the response to the low and high doses might be different, only one dose of irradiation was usually used in the aforementioned studies. For the first time we investigated induction of PFG after irradiation in wide dose range. Our study focused on G0 non-proliferated cells; therefore effect of IR was tested without modification of the cell properties such as proliferation of HSPC by growth factors. Even though we observed PFG induction in irradiated samples, we also detected PFG in control samples. Finally, we observed very low, but statistically significant increase of BCR-ABL PFG in cells irradiated with low doses compared to controls. The likely reason for absence of such effects at higher dose is onset of apoptosis, which is usually evident at the doses > 50 cGy^32^. We also did not reveal any increase of copy number in the irradiated cells compared to controls, as shown in aforementioned studies with cell lines. Likely explanation could be that our cells were in G0 phase and multiplication of such cells with PFG could not occur. Thus, it is tempting to speculate that cell cycle stimulation should be validated in further studies of radiation-induced PFG in hematopoietic cells.

## Materials and methods

### Ethical considerations

This study has been approved by the Ethics Committee of Children’s Hospital in Bratislava and all experiments and methods were performed in line with relevant guidelines. All UCB samples were provided with an informed consent from a parent or a legal guardian for study participation.

### Cells

Mononuclear cells were extracted from UCB as previously described^19^ and frozen in liquid nitrogen or freshly used. For analysis of PFG by RT-qPCR, γH2AX fluorescence, and apoptosis by flow cytometry we used frozen cells. Frozen MNC samples have been chosen for experiments based on results of our previous study, where these MNC were tested by RT-qPCR as negative^11^. After fast thawing of MNC in water bath at 37 °C, adherent monocytes were removed by 2-h incubation in RPMI medium supplemented with 10% FBS, 100 IU/ml penicillin and 100 µg/ml streptomycin, under standard incubation condition. Otherwise, for quantification of DNA repair foci by fluorescence microscopy and DNA damage response by comet assay freezing/thawing step was omitted and freshly extracted UCB MNC were immune-magnetically separated into CD34+ HSPC and CD34− lymphocytes using CD34 MicroBead Kit (Miltenyi Biotec, Bergisch Gladbach, Germany) and subsequently irradiated.

### Cell irradiation

The cells were irradiated on ice by γ-rays at the dose rate of 0.35 Gy/min using a THERATRON Elite 80 (MDS Nordion, Ottawa, Canada). During irradiation the cells were either in 25 ml tissue culture flasks (TPP, Switzerland) or on microscopic slides for the comet assay (ThermoShandon, Pittsburg, PA, USA) in concentration of 2 × 10^6^ cells/ml and 2.5–3 × 10^4^ cells/slide, respectively. The irradiated cells were briefly warmed to 37 °C in a water bath and then incubated at 37 °C in a CO_2_-incubator till analysis. Sham-irradiated control cells were concurrently subjected to the same manipulations as irradiated ones.

### Apoptosis

The MNC were harvested in different time points after irradiation (3, 24 or 48 h), spun down 100 g/10 min, washed in PBS and resuspended in 100 µl of the Annexin kit buffer (Roche, Basel, Switzerland) and analyzed for apoptosis by flow cytometry. Anti-human CD45-V450 antibody 1:50 (100 µg/ml, BD biosciences, San Jose, California, USA) was used to distinguish nuclear cells from erythrocytes and anti-human CD34-APC antibody 1:20 (Miltenyi Biotec) for gating HSPC. In the second part of apoptosis experiments anti-human CD19-AmCyan antibody 1:15 (25 µg/ml, BD biosciences) was used to specify B-cell precursors and anti-human CD38-PE-Cy7 antibody 1:20 (25 µg/ml, BD biosciences) to identify CD34+CD38+ progenitor cells and CD34+CD38− HSC/MPP. Annexin-V (BD biosciences) and 7AAD (BD biosciences) were used to analyze apoptotic and late apoptotic/necrotic cells (LAN), respectively. Cell population positive for Annexin-V staining, but negative for 7-AAD were gated as apoptotic. LAN cells were characterized as positive for both Annexin and 7-AAD (Supplementary Figure 3). Cells were incubated with specific antibodies for 30 min. All data were acquired with a FACSCanto II cytometer (BD biosciences) and analyzed using FACSDiva 6.0 software (BD biosciences). Approximately 5 × 10^5^ CD45+ MNC cells were measured and part of them 0.5–2% (≈ 500 cells) accounted for CD34+ HSPC. CD34+CD38+ progenitor cells represented approximately 90% of CD34+ HSPC cells and 10% accounted for CD34+CD38− population that is enriched with hematopoietic stem cells (HSC).

### Evaluation of DSB by 53BP/γH2AX immunostaining and automated fluorescent microscopy

The samples for enumeration of DNA repair foci were processed essentially as previously described^21^. Each sample was spread and analyzed on two fields of microscopic cytoslide. Approximately 200,000 of cells were cytospined on each field. A primary antibody mix consisted of 53BP1 polyclonal/rabbit antibody at 1:800 dilution and γH2AX monoclonal/mouse antibody (both antibodies from Novus biologicals) at 1:400 dilution. The secondary antibody mix consisted of Alexa Fluor 488 IgG (H1L) anti-rabbit, 1:200, and Alexa Fluor 555 IgG (H1L) anti-mouse, 1:200 (Life technologies, Molecular probes, Eugene, OR). Upon immunostaining, Vectashield mounting medium (Vector Laboratories, Peterborough, United Kingdom) was dropped on microscopic cytoslides, and cover slips (Menzel-Gläser, Germany) were sealed using a translucent nail polish. Image acquisition was conducted using the Metafer slide scanning system 3.6 (MetaSystems GmbH, Germany) incorporated to the Zeiss AxioImager.Z2 fluorescence microscope (Carl Zeiss Microscopy, Germany) (Supplementary Figure 4). The main parameters of acquisition were objective magnification 63× , the number of focus planes 11, and the focus plane distance 28/40 µm. For image analysis in 250 cells per exposure condition we used JCountPro software as was previously described^33^.

### Analysis of DNA damage response by γH2AX fluorescence

γH2AX fluorescence in the place of DNA breaks, including cells expressing whole nucleus γH2AX staining (so called γH2AX pan-staining)^21,34^ was measured using flow cytometry. Approximately 3 million of cells were harvested 0.5, 2 and 18 h after irradiation. The cells were spun down 10 min/150 g. Then the cell pellet was resuspended in 1 ml of 4% paraformaldehyde in PBS and incubated at room temperature for 10 min. After incubation, 3 ml of PBS was added to the sample and centrifuged again at 15 min/150 g. Then 500 µl of 70% ethanol was gently added to the cell pellet and incubated 30 min. The cells were firstly rehydrated on ice using a buffer solution containing 1% BSA and 0.1% Triton X-100 in PBS and then stained in 100 µl PBS by specific antibodies. To analyze fluorescence emission of γH2AX, anti-γH2AX-Alexa Fluor 488 conjugate (BD biosciences) was used in concentration 1:15. To distinguish different cell populations, anti-human CD45-V450 1:50, CD34-APC 1:15, and CD38-Pe-Cy7 1:20 (BD biosciences) were used. Finally, the cells were resuspended in 200 µl of PBS and γH2AX fluorescence was analyzed using FACS Canto II (BD biosciences) flow cytometer.

### Comet assay

DNA damage response was detected in parallel by alkaline^35^ and neutral single-cell gel electrophoresis (SCGE)^36^ also called comet assay. Briefly, suspensions of the probands cells in 0.75% LMP agarose dissolved in PBS was spread onto microscopic slides pre-coated with 1% NMP agarose. About 25,000 cells were putted on slide. After irradiation, the cells were lysed for 1 h at 4 °C in a buffer consisting of 2.5 M NaCl, 0.1 M Na_2_EDTA, 10 mM Tris–HCl and 1% Triton X-100, pH = 10. After the lysis, the slides were placed in an electrophoresis box for DNA unwinding for 40 min in the electrophoretic solution (0.3 M NaOH and 1 mM Na_2_EDTA, pH ≥ 13). Electrophoresis was conducted at temperature of 4 °C for 30 min at the voltage of 0.73 V/cm, with accompanying amperage of ~ 300 mA. The slides were then neutralized in 0.4 M Tris–HCl, drained, stained with 5 µg/ml ethidium bromide (EtBr) and covered with cover slips. In the neutral comet assay, lysis was performed in the alkaline lysis solution supplemented with 2% sarcosyl. Unwinding (20 min at 4 °C) and electrophoresis (60 min at the voltage of 0.41 V/cm, with an accompanying amperage of ~ 50 mA) steps were carried out in electrophoretic buffer consisting of 0.1 M Tris–HCl and 0.3 M sodium acetate at pH = 9. At least one hundred of EtBr-stained nucleoids per proband in one electrophoresis run were examined with a Carl Zeiss AxioImager.Z2 fluorescence microscope using a computerized image analysis Metafer 5 (MetaSystems GmbH, Germany) (Supplementary Figure 5). The percentage of DNA in the tail and tail moment was used as a parameter for the measurement of DNA damage in alkaline and neutral SCGE, respectively.

### RNA isolation and cDNA synthesis

Total RNA was isolated with RNAzol (Research Molecular Center, Ohio, USA) using standard protocol recommended by the manufacturer from 10 million of cells at 3 h and 24 h post-irradiation^10^. RNA yield was measured by Nanodrop (Thermo Scientific, St. Leon-Rot, Germany). cDNA was synthesized by reverse transcription of total RNA in the standard reaction containing 1–3 μg of total RNA following the manufacturer’s protocol (Thermo Scientific) and then used as a template for RT-qPCR.

### Real time quantitative PCR

The RT-qPCR was performed as previously described^10^. Briefly, the RT-qPCR contained 2 µl cDNA (150 ng RNA equivalent), 300 nM each primer (VBC-Biotech, Wien, Austria), 200 nM probe (5′-fluorophore was FAM, 3′-quencher was BHQ1; (Merck, Darmstadt, Germany)), and HOT FIREPol Probe qPCR mix from Solis BioDyne (Tartu, Estonia). The plasmid standards with individual fusion genes were synthesized by Ipsogen (Qiagen, Marseille, France). Protocol of RT-qPCR, the primers and the probes were designed according to Gabert et al.^37^. RT-qPCR was performed on a BioRad CFX96 instrument. All samples were run in triplicate and regarded as positive if at least one of three tested tubes was positive.

### Sequencing of the RT-qPCR product

The sequencing of the RT-qPCR product was performed as we previously described^11^. Briefly, the RT-qPCR product was digested with Exo/Sap (Affymetrix, California, USA) to remove all the contaminating primers, TaqMan probe and dNTPs. The RT-qPCR product was firstly re-amplified in standard PCR using primers that contained restriction sites allowing directed subcloning of the PCR product into a sequencing vector pUC18. After the subcloning step and transformation of competent cells DH5α (RbCl^+^), recombinant plasmid DNA was isolated and employed as a template in the sequencing reaction using sequencing primers of the vector.

### Quantification and statistical analysis

Statistical analysis was carried out by Statistica 8.0 software (Dell software, Round Rock, Texas, United States). The data were analyzed by ANOVA with Fisher LSD or t-test and the results were considered significantly different at p < 0.05. Correlation between PFG positivity, apoptosis or RNA yield was analyzed by the Spearman correlation test. If the p value was lower than 1 × 10^–6^ we displayed the p value as p < 0.000001, in other cases we provided exact numbers.

## Supplementary information

Supplementary Legends.

Supplementary Figure S1.

Supplementary Figure S2.

Supplementary Figure S3.

Supplementary Figure S4.

Supplementary Figure S5.

## Data Availability

The authors report that data could be available upon request.
