# Supplementary material for: DNA damage response and preleukemic fusion genes induced by ionizing radiation in umbilical cord blood hematopoietic stem cells
Source: Sci Rep. 2020 Aug 24;10:13722. doi: 10.1038/s41598-020-70657-z (PMC7445283; doi:10.1038/s41598-020-70657-z)
Supplement: Supplementary file 1 — Supplementary Legends. [file 41598_2020_70657_MOESM1_ESM.docx]

**Supplementary Figure 1:** Apoptosis in CD34+CD38+ progenitor and CD34+CD38- HSC/MPP. Figure shows percentage of A) early apoptotic cells, B) LAN cells in CD34- lymphocytes, CD34+CD38+ progenitors and CD34+CD38- HSC/MPP at different time points post-irradiation with doses 0, 10, and 200 cGy. Mean value and 95% confidence interval is shown from 6 samples for 200 cGy, and 3 samples for 10 cGy.

**Supplementary Figure 2:** Apoptosis in CD34- lymphocytes and CD34+ HSPC by combining the data from HSC/MPP and progenitors shown in Figure 2 with the data presented in Figure 1. Figure shows percentage of live cells in CD34-lymphocytes and CD34+ HSPC at different time points post-irradiation with the dose of 0, 10, 200 cGy. Mean value and 95% confidence interval is shown in each data point.

**Supplementary Figure 3:** Representative figure of gating strategy for analysis of apoptosis by flow cytometry. In sequence, single cells, CD45+ lymphocytes (green), CD34+ HSPC (black), CD34+CD38+ progenitors (yellow) and CD34+CD38- HSC/MPP (dark green) were gated. Next, the Annexin V- (FITC-A) and 7AAD- (PerCP-A) live cells were gated using cross gating for tested populations.

**Supplementary Figure 4:** Representative image of DNA repair foci. By red color γH2AX foci, 53BP1 foci and their co-localization are visualized in red, green, and yellow, correspondently.

**Supplementary Figure 5:** Representative figure of comets. A) Undamaged UCB cells. B) Damaged UCB cells.
