# Supplementary figures and images for: DNA damage response and preleukemic fusion genes induced by ionizing radiation in umbilical cord blood hematopoietic stem cells
Source: Sci Rep. 2020 Aug 24;10:13722. doi: 10.1038/s41598-020-70657-z (PMC7445283; doi:10.1038/s41598-020-70657-z)

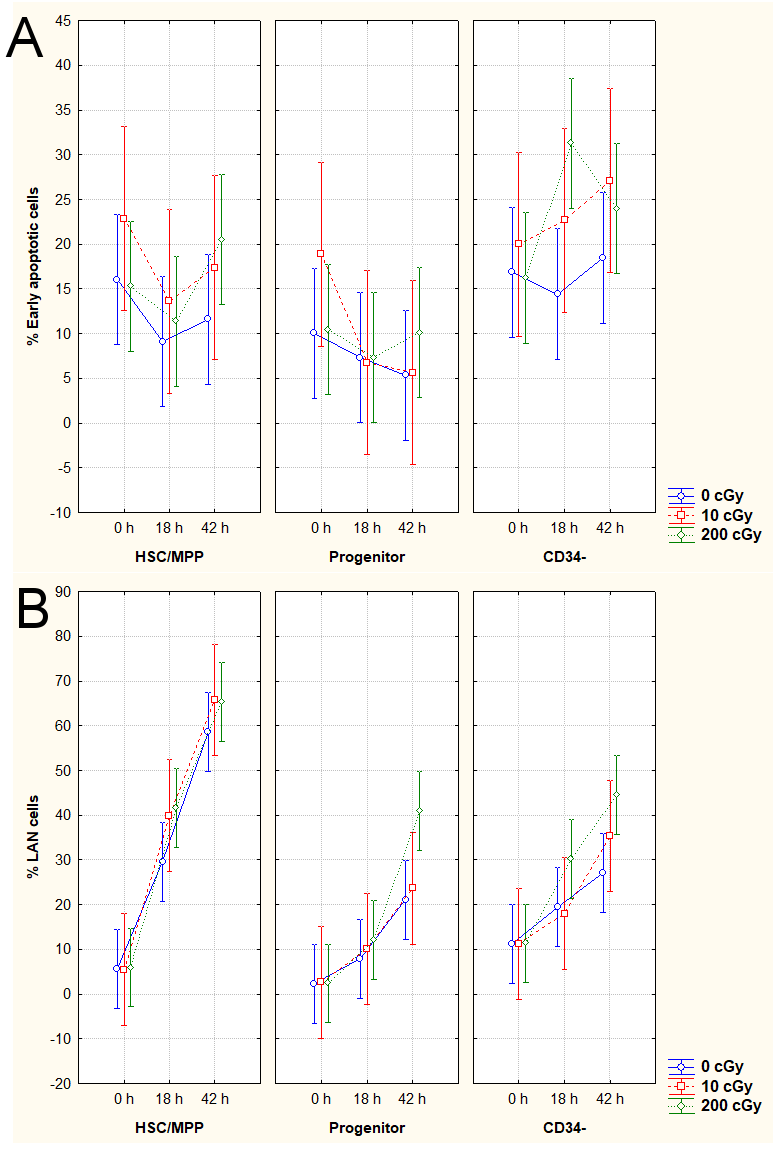

Supplement: Supplementary file 2 — Supplementary Figure S1. [file 41598_2020_70657_MOESM2_ESM.tif]

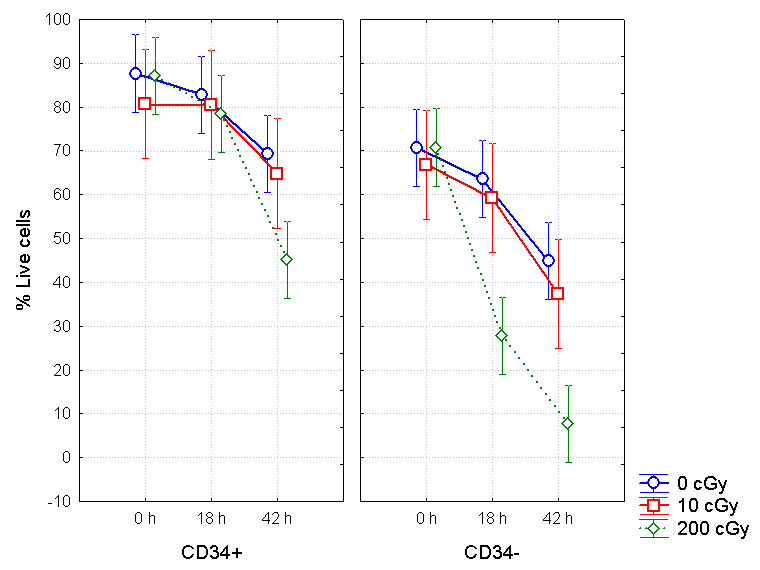

Supplement: Supplementary file 3 — Supplementary Figure S2. [file 41598_2020_70657_MOESM3_ESM.tif]

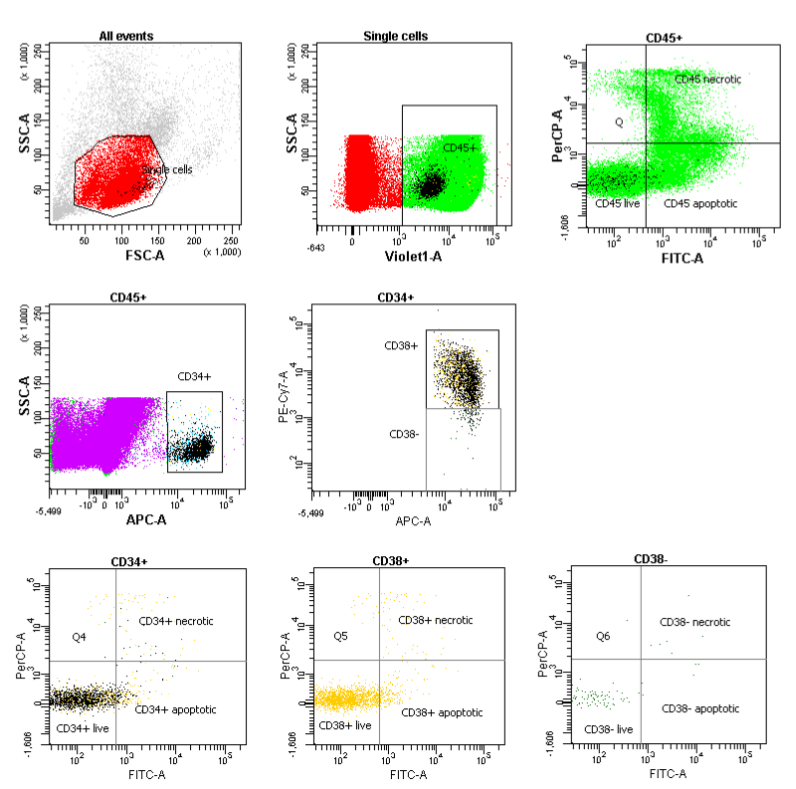

Supplement: Supplementary file 4 — Supplementary Figure S3. [file 41598_2020_70657_MOESM4_ESM.jpg]

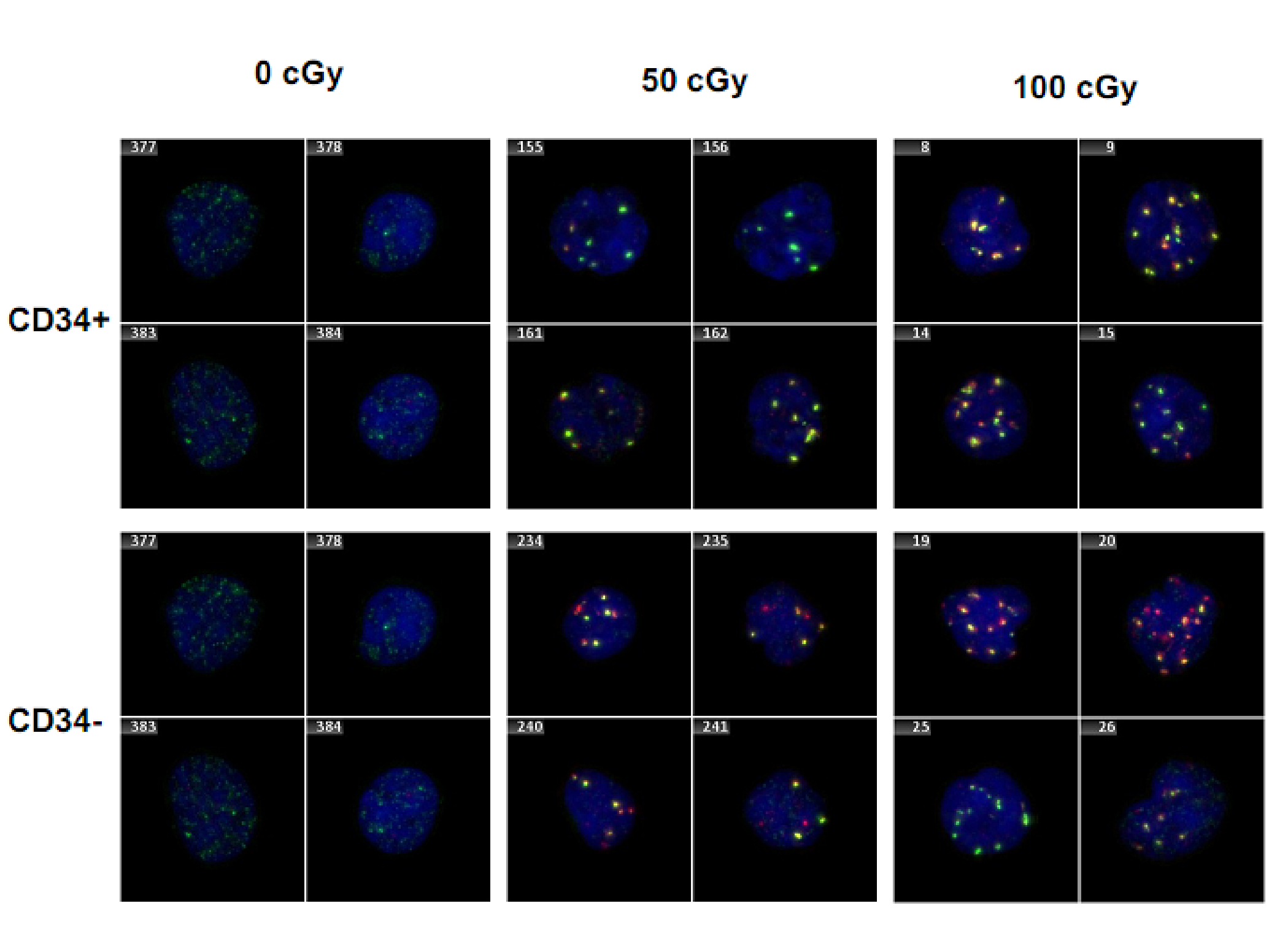

Supplement: Supplementary file 5 — Supplementary Figure S4. [file 41598_2020_70657_MOESM5_ESM.tif]

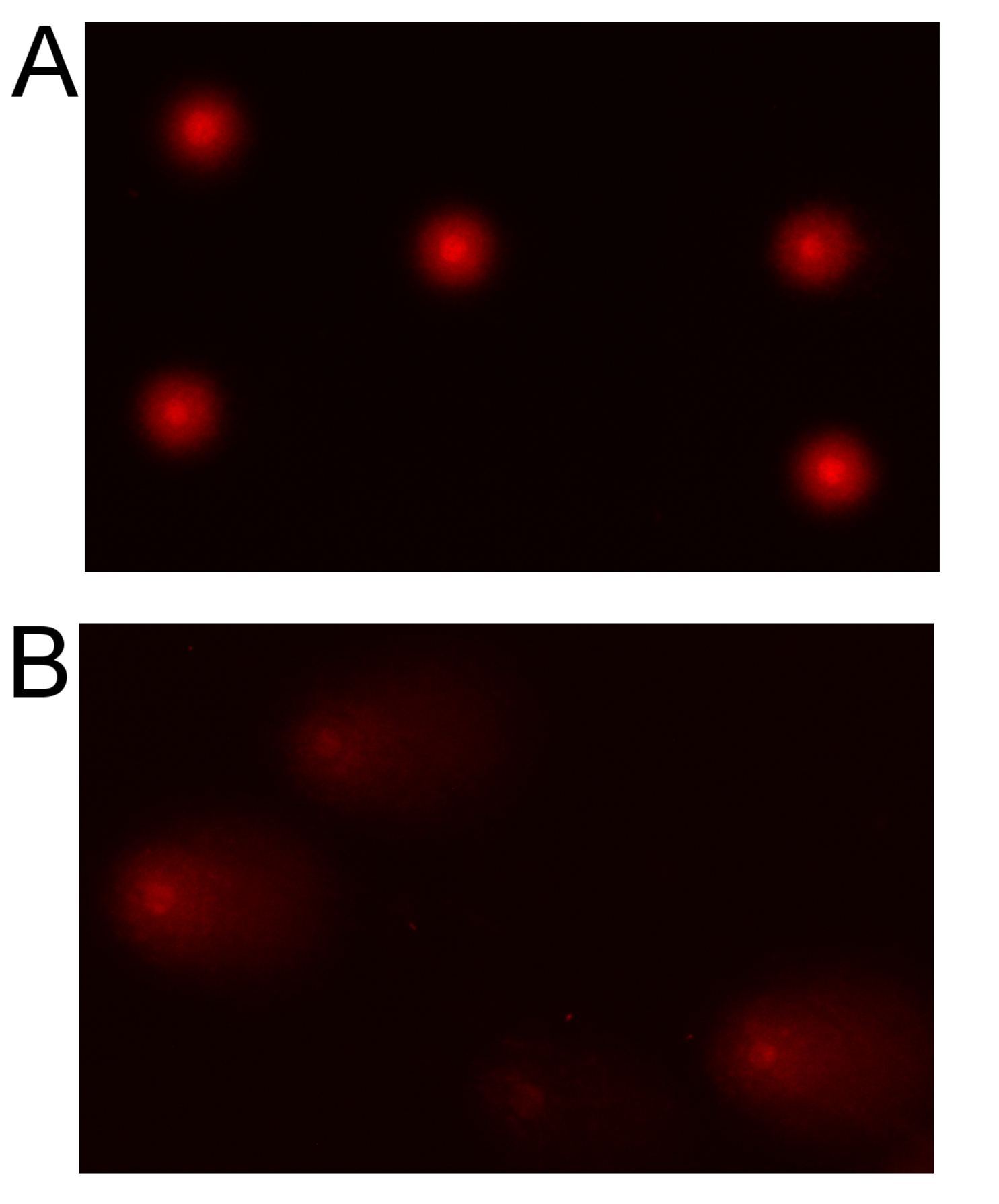

Supplement: Supplementary file 6 — Supplementary Figure S5. [file 41598_2020_70657_MOESM6_ESM.jpg]
